# Tuning Colloidal Gel Properties: The Influence of Central and Noncentral Forces

**DOI:** 10.1021/acs.langmuir.4c03602

**Published:** 2025-01-28

**Authors:** Florence J. Müller, Shivaprakash N. Ramakrishna, Lucio Isa, Jan Vermant

**Affiliations:** Department of Materials, ETH Zurich, 8093 Zurich, Switzerland

## Abstract

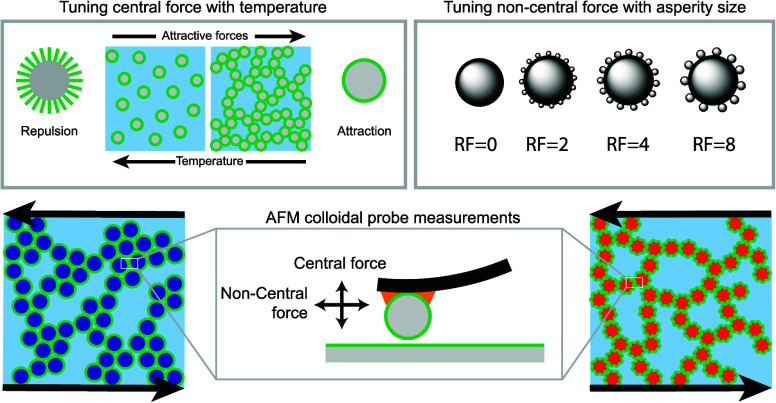

Colloidal gels, ubiquitous in industrial applications, can undergo reversible solid-to-liquid transitions. Recent work demonstrates that adding surface roughness to primary particles enhances the toughness and influences the self-healing properties of colloidal gels. In the present work, we first use colloidal probe atomic force microscopy (CP-AFM) to assess the quantitative changes in adhesive and frictional forces between thermoresponsive particles as a function of their roughness. The presence of static friction, generated by interparticle adhesion results in noncentral forces, leading to network structures that are more readily constrained in their nodes. Systems with higher friction exhibited increased sedimentation stability, a decrease in percolation threshold and a more abrupt elastic to plastic transition, but an enhanced capacity in storing elastic energy until fluidification. Additional experiments with geometrically smooth but “chemically rough” (patchy) particles further emphasized the importance of static interparticle friction in the macroscopic yielding and recovery behavior of colloidal gels.

## Introduction

Colloidal suspensions are ubiquitous systems, which can for instance be found in nature as mud, in construction as cement or in a variety of consumer products. In all of these systems, the interparticle interactions dictate the macroscopic behavior of the material, which is why their implications need to be understood.^1,2^ Most studied interparticle forces, even if possessing different origins such as electrostatics, van der Waals (vdW) or steric interactions,^3^ are nonetheless categorized as central forces, indicating that their force vectors pass through the centers of mass of the particles and they can typically be described by a pairwise potential that only depends on interparticle distance. However, when particles come into contact, i.e., when forming “bonds” in a colloidal gel, additional noncentral forces, such as frictional forces, come into play.^4,5^ Particles in the gel network experience these noncentral forces when tangential stresses and relative motion between the particles occur without a necessary change of the separation of their centers of mass.

These noncentral, frictional forces depend on the topography and the contact area between the particles, as well as on the physicochemical properties of the particles’ surfaces. Understanding the correlation between contact forces and bulk behavior has been emerging in recent literature. For example, contact forces and macroscopic aging in particle suspensions have been studied using optical tweezers to create and deform linear aggregates under twisting and bending.^6−9^ Alternatively, colloidal probe atomic force microscopy (CP-AFM) has been used to measure single-particle interactions during contact and relative sliding, clearly identifying normal (central) and tangential (noncentral) force components.^10,11^ While both methods offer precious insights, it is worth noting that the AFM cantilevers offer the advantage of applying forces that are orders of magnitude greater than those attainable with optical tweezers.^12^

Among the different properties inducing the emergence of noncentral forces, surface roughness has been more widely studied. For instance, in shear-thickening suspensions, it has been extensively shown that surface roughness has a significant impact on the maximum packing fraction and the rheology, where the onset of discontinuous shear thickening occurs at lower shear rates in rougher systems.^5,10,11,13,14^ Recently, it has been shown that surface roughness can also increase the strength of capillary suspensions.^15^ However, physical surface roughness is not the only way to induce noncentral forces in a particle system: surface chemistry and in particular chemical heterogeneities, such as patchiness on the particle surface, can also influence their self-assembly behavior^16−19^ and the stability of colloidal materials.^20−24^

Most work to date has focused on dense, nearly jammed suspensions,^16,25−27^ but in structurally more open colloidal networks which occur at lower volume fractions, noncentral forces are expected to strongly influence how the network stores energy,^28^ as well as how the colloidal gel rearranges under stress. In nonfrictional systems with only central attractive interactions, elasticity essentially follows Cauchy-Born theory, arising from a few weak connections between locally isostatic clusters.^29^ Simulations indicate that elasticity and the transition to plastic flow are influenced by stress localization and network restructuring, where increased deformation leads to localized stretching and alignment of network chains, ultimately causing yielding through the breaking of a few critical bonds.^30^ Further numerical studies elucidated that increasing attraction strength enhances both the networks’ elasticity and resilience, making the structure less sensitive to the loss of interparticle bonds.^31^ Increasing the attraction range was shown to influence elasticity, with extended ranges leading to coarser structures and reduced rigidity, as particle clusters become less interconnected and more prone to reorganization under stress.^32^ These studies focused primarily on central forces, but surface heterogeneities, both chemical and physical, have been suggested to significantly impact the rheological properties and gelation mechanics of colloidal gels in both simulations^22^ and experiments.^28^ In the present work, we aim to clarify these effects of surface roughness and patchiness, and how adhesive central forces and frictional noncentral forces influence the yielding transition in colloidal gels at low volume fractions.

Using thermoreversible rough and patchy systems, we keep control over the kinetic pathway of gelation, and ensure that the systems start from an initially comparable structure. We carry out systematic CP-AFM experiments, using the same particles used to form the gels, under identical conditions in terms of temperature and solvent quality, allowing us to measure adhesion and gain initial insights into the frictional forces experienced between the particles. We compare these single-particle properties to the macroscopic properties of the corresponding gels, focusing particularly on the transition from elastic to plastic flow or fracture.

The model system used in this work is tunable in terms of surface topography, patchiness and interaction potential. We synthesize silica particles of varying surface roughness using an electrostatic heteroaggregation approach^33^ by adsorbing different sizes of asperity particles to larger core particles. Silica particles of different roughnesses are then grafted with a brush of octadecyl, which makes them undergo a change in interaction when suspended in tetradecane as a function of temperature.^34^ The brush is solvated at higher temperatures, which makes the particles repulsive. At temperatures below 20 °C, the interparticle forces are attractive, and the magnitude of the force depends on the temperature. To gain further insight on the role of surface heterogeneity, we also examine chemically patchy particles decorated with discrete islands of octadecyl. Chemical patchiness induces a similar noncentral force between adhesive particles without the need for geometrical interlocking provided by topographical roughness. This combination of precision synthesis as well as complementary single-particle and macroscopic characterization allows us to shed new light on the complex interplay between central and noncentral forces in the yielding of colloidal gels and develop guidelines to design systems with targeted properties. This study systematically investigates the influence of surface topography (noncentral force) as well as vdW adhesion interactions (central force) on how the material yields and transitions from an elastic solid gel to a plastically flowing or fracturing material.

## Materials and Methods

### Materials

Ethanol (99.8%, Merck), tetraethyl orthosilicate (TEOS, 99%, Sigma-Aldrich), Triethyl(octadecyl)silane (TMOS, technical grade, Sigma-Aldrich), ammonia solution (NH_4_OH, 25%, Merck), polydiallyldimethylammonium chloride (Poly-DADMAC, 400–500 kDa, 20 wt %, Sigma-Aldrich), trimethoxy[3-(methylamino)propyl]silane (MAPTMS, 97%, Sigma-Aldrich), 1-octadecanol (octadecanol, 99%, Sigma-Aldrich), toluene (99.85%, Fisher Scientific), toluenesulfonic acid-p monohydrate (pTsOH, 98%, Sigma-Aldrich), propiolic acid (96%, Sigma-Aldrich), isopropanol (technical grade), deionized water (Milli-Q, Merck-Millipore), and hydrochloric acid (HCL, Sigma-Aldrich). All products were used as received.

### Particle Synthesis

Silica core particles of 750 nm in diameter were synthesized using the Stöber method. Solution II, consisting of 50 mL ethanol and 10 mL ammonia solution was stirred (500 rpm) in a 250 mL round-bottom flask and sealed with a rubber stopper to avoid evaporation. Solution I, consisting of 40 mL ethanol and 6.6 mL TEOS was prepared separately. 40 mL of solution I was then added to solution II at 50 μL/min using a syringe pump, where the feeding tube was immersed in solution II in order to decrease the concentration gradient. The reaction was stirred for 24 h before cleaning the particles 3 times with ethanol and 3 times with Milli-Q.

In order to make the raspberry particles, the cores were first rendered positive. To this end, 0.5 mL of Poly-DADMAC was dissolved in 400 mL of Milli-Q and stirred until fully dissolved. The core particles (1.5 g) were added to 100 mL of the Poly-DADMAC solution and stirred for 1h, before cleaning the particles 3 times with MiliQ and then exchanging the solvent for ethanol. The negatively charged berry particles were then added to the now positively charged core particles in the proportions indicated in Table S1. The assembly was then coated by adding a layer of MAPTMS (see amount for every particle roughness in Table S1), in order to covalently attach the berry particles to the core and provide a functional secondary amine outer layer. The particles were then cleaned in ethanol 3 times and the solvent was exchanged for isopropanol. The octadecyl layer was grafted through a click-like reaction, where a certain amount (see Table S1) of the prior functionalized octadecane-alkynoate was mixed with the particles in isopropanol at 40 °C overnight. The particles were then cleaned 3 times with isopropanol to remove excess reagent and dried in the vacuum oven.

Patchy SiO_2_ particles were synthesized using the Stöber method by adding 12.4 mL of TEOS to a solution of 200 mL ethanol, 18 mL H_2_O and 9 mL of ammonia solution in a 500 mL glass bottle using a dumbbell stirrer. A 5 v% solution of Triethyl(octadecyl)silane (TMOS) in ethanol (0.95 mL) was then injected to the suspension without a cleaning step at a rate of 2 mL/h and left to react overnight. The particles were then cleaned in ethanol three times and dried using a rotary evaporator and a vacuum oven at 30 °C for 48 h. The gel was formed by adding the particles to tetradecane and resuspending the particles using a vortex mixer and ultrasonication at 40 °C.

### Octadecane-Alkynoate Functionalization

The octadecanol was functionalized with an alkynoate group, following the Fischer esterification process. For this, 10 g (1 equiv) of octadecanol was dissolved in 70 mL of toluene at 50 °C in a 100 mL round-bottom flask. After complete dissolution, 0.5 g (5% of octadecanol weight) of pTsOH was added to the reaction, followed by 2.52 mL (1.1 equiv) of propiolic acid. A Dean–Stark trap and a condenser was then installed on the round-bottom flask, and the solution was heated to 135 °C to start the reaction, which was left for 24 h. The octadecane-alkynoate was purified by evaporating the toluene, the product was then dissolved in 20 mL of acetone and dropped into iced MiliQ for the octadecane-alkynoate to precipitate. The suspension was then isolated using a vacuum filter and dried under vacuum at 30 °C for 24 h. The product was stored in the freezer to avoid degradation.

### Functionalization of Glass

Glass fixtures such as glass slides for the AFM substrate and the rheology measurements as well as the cuvettes for the sedimentation experiments were functionalized with octadecyl. To that end, the flat glass slides were cleaned with plasma and the cuvettes were immersed overnight in a 1:1 solution of HCL and isopropanol. The samples were then immersed in ethanol and 1 mL of 5 v% of T-MAPS in ethanol solution was added to every 100 mL of ethanol used for the functionalization. After 3 h, the samples were cleaned by flushing them with isopropanol and then reimmersed in isopropanol. A 5 w/v% solution of octadecane-alkynoate in isopropanol was added in a ratio of 1 mL for every 100 mL of isopropanol. The container holding the samples was placed in a 40 °C warm bath for 3 h for the duration of the reaction. The samples were then cleaned with isopropanol and dried before further use.

### AFM Tribology Experiments

Adhesion and friction measurements were carried out by Bruker Dimension Icon AFM. Octadecyl-functionalized silica particles of varying roughness were glued to the tipless Au-coated cantilevers (CSC-38, Mikromash, Bulgaria) using the custom-made micromanipulator. Normal and torsional spring constants of the cantilevers were determined using Sader’s method.^35^ Surfaces having the same berry sizes as the colloidal probe were prepared by electrostatic heteroaggregation (analogous to the particle synthesis). The measurements were conducted by immersing both the colloidal probe and the surface in a custom-made temperature cell (see Figure S5) containing tetradecane solution. The temperature was gradually increased from 5.5 °C until 40 °C in an interval of 5 °C using the custom-made heater-cooler system. Before each measurements, we allowed a waiting of about 20 min for stabilization, and maintained an accuracy of ±1 °C. Adhesion measurements were conducted by ramping the Z scanner over a distance of 1 μm at a scanning rate of 0.25 Hz to acquire the force-vs-distance curves. About 128 force curves were collected from two distinct spots on the surface, selecting an area of 5 × 5 μm using the force volume map. The measured adhesion forces were then averaged.

Friction measurements were conducted by scanning the cantilever laterally over the sample surface and collected the friction loops over 2 distinct spots to calculate the average friction values. The slow scan axis was disabled and an aspect ratio of 20 × 20 was selected to acquire both trace and retrace images of the friction channel. A scan distance of 5 μm and a scan rate of 1 Hz was used for the friction measurements. The average friction force value was calculated by using the image math option in the Bruker nanoscope analysis software by subtracting the retrace image by trace image and dividing it by 2. The normal force was increased in an increment of 10 nN up to 70 nN and plotted as friction force vs normal force. The coefficient of friction values were then obtained by the slopes of friction force vs normal force curves.^36^ Lateral-force calibration was conducted using the thermal noise based method described by Mullin et al.^37^ More details are provided in the SI.

### Sedimentation Experiments

The sedimentation experiments were performed in cylindrical 10 mm × 75 mm borosilicate cuvettes, that were functionalized with octadecyl using the method described above. The cuvettes were immersed in a water bath, that was kept at a controlled temperature (5.5 °C) using a secondary water cycle. The advancement of the sedimentation front was observed using a Thorlabs zelux camera (CS165MU/M) that took timelaps images every 5 min. Image analysis was used in order to assess the sedimentation front.

### Patchy Particle Synthesis

The SiO_2_ particles were synthesized using the Stöber method by adding 12.4 mL of TEOS to a solution of 200 mL ethanol, 18 mL H_2_O, and 9 mL of ammonia solution in a 500 mL glass bottle using a dumbbell stirrer. A 5 vol % solution of triethyl(octadecyl)silane (TMOS) in ethanol (0.95 mL) was then injected to the suspension without a cleaning step at a rate of 2 mL/h and left to react over night. The particles were then cleaned in ethanol three times and dried using a rotary evaporator and a vacuum oven at 30 °C for 48 h. The gel was formed by adding the particles to tetradecane and re-suspending the particles using a vortex mixer and ultrasonication at 40 °C.

### Rheology Experiments

Rheology measurements were performed on an Anton Paar MCR 502 using a 22 mm plate–plate geometry. In order to avoid slip, a 22 mm borosilicate glass slide was functionalized using the method described above and glued to a disposable geometry of a diameter of the same diameter using UV glue. For the bottom plate, a disposable bottom plate was fitted with an octadecyl-functionalized glass slide of a diameter of 35 mm. The sample was loaded at 40 °C, cooled down to 5.5 °C and equilibrated for 15 min. Between the measurements, the gel was rejuvenated by cycling the temperature to 40 °C for 10 min and cooled to the investigated temperature for 15 min. Experiments to determine the percolation threshold were carried out by forming gels at low volume fractions and measuring a strain amplitude sweep at 1 rad/s at 5.5 °C. The systems were estimated to be at percolation when the measurement showed a measurable modulus with a linear viscoelastic response region for more than half a decade of strain amplitude and any positive volume fraction increment would show an even larger viscoelastic region, while any volume fraction reduction did not show clear linear viscoelasticity, i.e., no discernible gelled behavior where *G*′ > *G*″. The stress amplitude sweep measurements were performed at 1 rad/s. Continuous creep measurements were performed for 5 min, followed by a stress relaxation step at 0 Pa for 5 min. The recoverable strain was calculated by taking the ratio of the plastically recovered strain (strain value after creep - strain value after recovery) over the strain after the creep experiment.

## Results and Discussion

**Figure 1:**
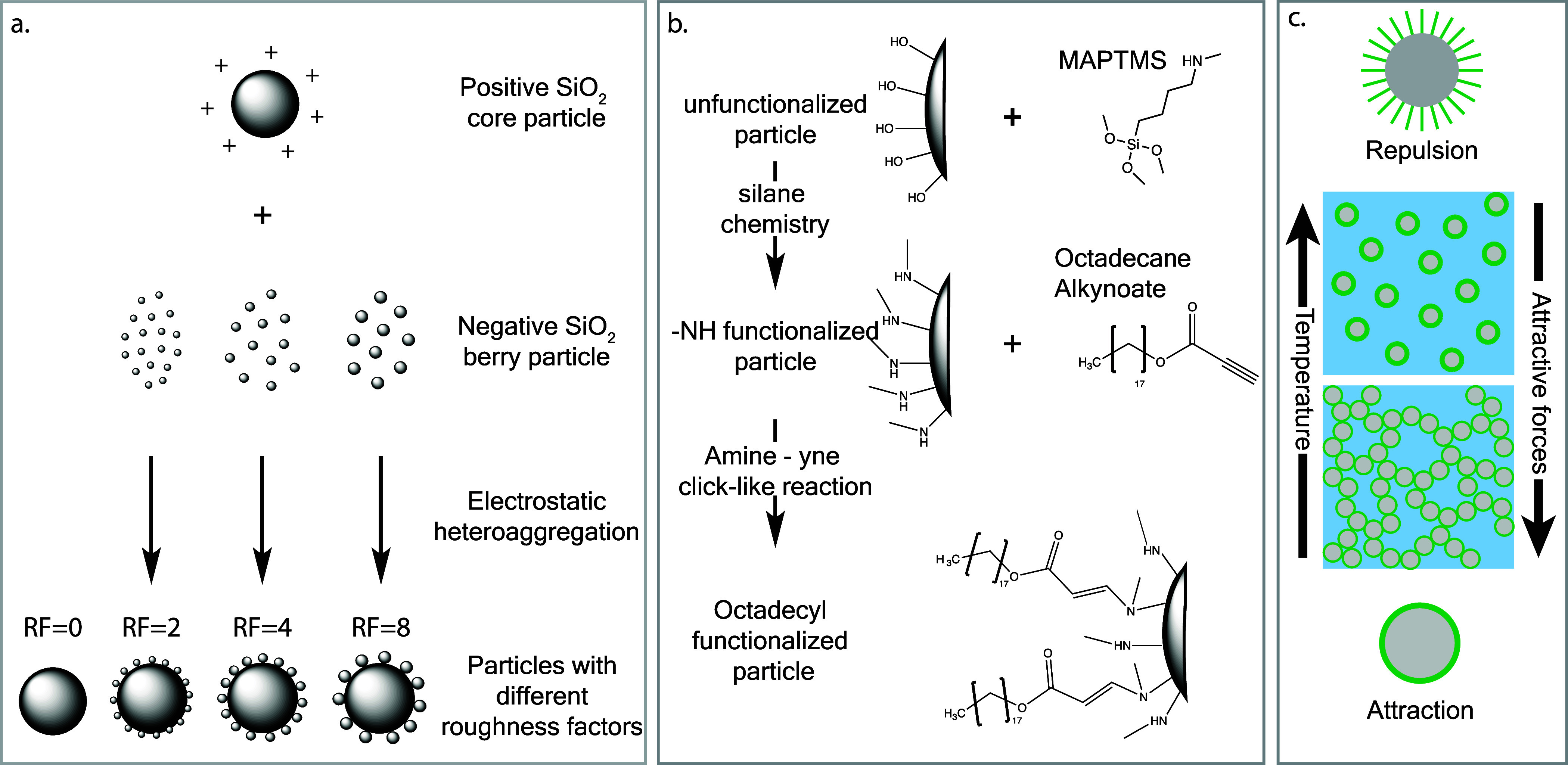
Model system of thermoresponsive silica core–shell particles of varying roughness. (a) Electrostatic heteroaggregation of asperity particles of different sizes to the core particle, (b) Grafting of the functionalized particles with secondary amines and subsequently with octadecane-alkynoate to form thermoresponsive silica particles, (c) Thermoresponsive gelation mechanism of the octdadecyl-functionalized silica particles suspended in tetradecane.

We prepared silica particles with varying degrees of roughness, as depicted in Figure 1a, adapting a previously published protocol^28^ (see Materials and Methods). This was achieved by attaching negatively charged “berry” or asperity particles of different sizes (15, 30, and 60 nm) onto positively charged core particles with a constant diameter of 750 nm, which are referred to hereafter as roughness factor RF = 2, RF = 4 and RF = 8, respectively. The roughness factor (RF) is defined as . Together with these particles, we also examined the smooth core particles without asperities, therefore having RF = 0. In the next step, we stabilized and solidified this assembly using a layer of Trimethoxy[3-(methylamino)propyl]silane (MAPTMS), which also introduced functional secondary amine groups to the particle surface (as illustrated in Figure 1b). We then proceeded to graft an octadecyl layer onto the particles through an efficient click-like chemistry process. This grafting step involved a reaction between the secondary amine (−NH) groups and the alkynoate group of a alkynoate-modified octadecane chain (see Materials and Methods). Additionally, patchy particles were fabricated by first synthesizing Stöber silica particles, followed by the slow addition of Trimethoxy(octadecyl)silane to induce a hydrolysis–condensation surface reaction. The amphiphilic nature of the molecule results in a patchy distribution of the grafting agent on the particle surface, as it can form micelles, adsorb to the particle, or create oligomers, leading to inhomogeneous coverage (see Figure S11). This patchy surface coverage has similar effects to physical surface roughness, inducing a noncentral interaction which can be viewed as a “chemical friction” (see Figure S12), which will be explored later in the manuscript.

Figure 1c illustrates the thermoreversible behavior of the octadecyl-grafted particles in tetradecane. Lowering the temperature induces van der Waals (vdW) interactions and adhesion upon particle-particle contact, leading to particle aggregation and gelation for sufficient volume fractions, as previously reported by Eberle et al.^34^ Conversely, at higher temperatures, steric repulsion between the brushes dominates, resulting in the sample transitioning into a macroscopic fluid state. Importantly, this system allows us to not only switch the attractive interactions on and off by varying the temperature between 20 °C and 5.5 °C, but it also permits the fine-tuning of the attractive interaction strength by controlling the temperature between this range. As a result, this colloidal system can form a nascent gel when cooled below 20 °C, circumventing any thixotropic effects associated with sample loading. Furthermore, varying the temperature within this range allows for the creation of gels with varying degrees of strength.

**Figure 2:**
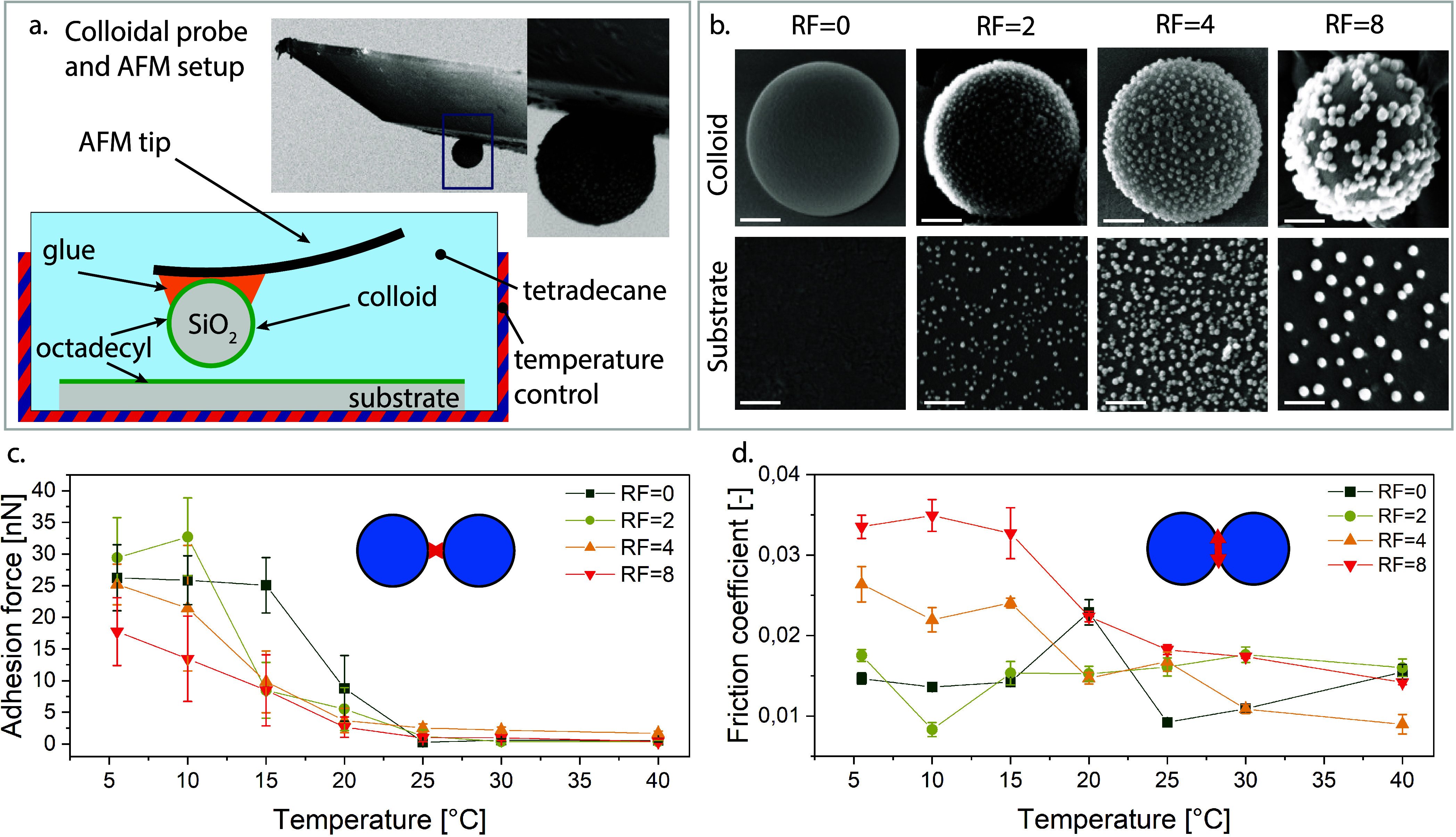
Colloidal probe AFM measurements. (a) Schematic of the experimental setup of the immersed colloidal probe, with a typical SEM image of the colloidal probe (particle diameter of 750 nm with RF = 2) SEM showing that the particles can be glued without affecting the surface asperities. (b) SEM images of the (i) colloids of varying roughness (ii) and of the substrates that mimic the same surface topography as the colloidal probes (scale bar = 200 nm). (c) Adhesion force for the different roughness factors at varying temperatures. (d) Friction coefficient for the different roughness factors at varying temperatures.

We conducted single-particle AFM experiments to quantitatively characterize individual particle properties and demonstrate the tunability of central and noncentral forces with temperature and surface roughness. In these experiments, all four particle types with the different roughness factors were affixed to the tips of AFM cantilevers, forming what is commonly referred to as a colloidal probe,^38^ as shown in Figure 2a. The SEM images show the successful attachment of the particles leaving their surface exposed in the contact region. To mimic the possible interlocking of particles via their surface asperities, we prepared a glass slide with the same asperity particles as those used for the colloidal probes (see Figure 2b, substrate). Subsequently, we applied the same octadecyl functionalization to these glass slides. We designed and constructed a custom temperature-controlled cell to replicate the bulk environment (temperature and suspending media) of two single particles in contact (see Figure S5). This cell allowed us to immerse both the substrate and the colloidal probe in the suspending medium fluid tetradecane, and accurately control the temperature with a secondary water cycle. In the adhesion experiments, we measure the normal force between the colloid and the substrate as a function of separation during approach and retraction.

The measurements for particles of varying roughness at different temperatures are presented in Figure 2c (force measurements in Figures S1–S3). Notably, for all samples, the magnitude of the adhesion force between the two surfaces decreases as the temperature increases. This observation confirms that central adhesion forces between particles can be varied from 0 to 35 nN by adjusting the temperature. It is worth mentioning that the adhesion forces of RF = 0 and RF = 2 are quite similar, whereas the rougher colloids (RF = 4 and RF = 8) exhibit lower adhesion forces compared to the smoother ones. This decrease in adhesion with increasing roughness has been experimentally observed by Ramakrishna et al.^39^ and aligns with the Rumpf model predictions^40,41^ for a smooth spherical particle of radius *R* in contact with a planar surface, separated by an asperity of radius *r*:
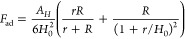
1Here, *H*_0_ is the distance of closest approach between surfaces. The first term describes the interaction between the large particle and the asperity, while the second term describes the interaction between the particle and the planar surface.^42^*A_H_* is the Hamaker constant, which depends on the refractive indices and dielectric constants of the silica, the octadecyl brush and the suspending medium tetradecane. As the coating is the same for all systems, *A_H_* can be assumed comparable for all samples.^41^ The refractive indices and dielectric constants of all three materials in this system depend on temperature and, according to the Lifshitz Theory,^43^*A_H_* will thus be temperature-dependent, and will increase with decreasing temperature. Additionally, as the temperature decreases, the brush solidifies, which eliminates the entropic elastic component of the steric interaction.^34^ Therefore, the increase in adhesion as temperature decreases stems from changes in *A_H_* and brush conformation. The decrease of the adhesion with increasing roughness is due to corresponding changes to the contact area and the distance between the particle and the substrate. One possible explanation is that the presence of asperities reduces the overall adhesion by increasing the average distance between the particle and the substrate. The extent of this effect is expected to be dependent on the topography of the substrate, as the nature of the contact during a single adhesion measurement can vary between asperity-asperity, asperity-flat, or flat–flat, depending on the orientation of the colloid on the probe and the surface.^44^ It is intuitive that, for small asperities, the adhesion approaches that of smooth particles, given the consistent surface chemistry for all samples.

To investigate the frictional behavior, the sliding friction coefficient as a function of temperature was measured by laterally moving the colloidal probe against substrates of matching roughness at different normal forces, see Figure 2d. The friction coefficient values were determined from the slope of friction force vs normal force curves, as shown in Figure S4. Although the surfaces exhibit lubricious behavior due to the octadecyl brushes, leading to low absolute values of the friction coefficients, we observe that friction is strongly influenced by adhesive interactions at the contact interface, increasing 2-fold between the smoothest (RF = 0) and roughest (RF = 8) conditions. For samples with RF = 8 and RF = 4, we detect an increased friction coefficient at low temperatures, which can be attributed to increased dissipation at contact in the presence of stronger adhesive force, responsible for bringing the particles into closer contact.^45^ However, as the temperature rises, repulsive interactions between the colloidal probe and the surface particle dominate, primarily due to steric interactions, leading to a decrease in friction. While there can be different coefficients for sliding or rolling friction, the presence of static friction emerges due to the adhesive contacts. Adhesion, manifesting as a finite lateral force at zero normal load, can also be expected to increase dynamic friction (both sliding and rolling). The data in Figure 2c,d show a clear correlation between adhesion at low temperatures and an increase in the sliding friction coefficient. Additionally, the effect of adhesion is compounded by surface topography, as described above. Overall, the AFM measurements provide a partial, yet consistent and internally coherent perspective on how topography affects both adhesion and friction, which we now proceed to relate to macroscopic rheological behavior.

**Figure 3:**
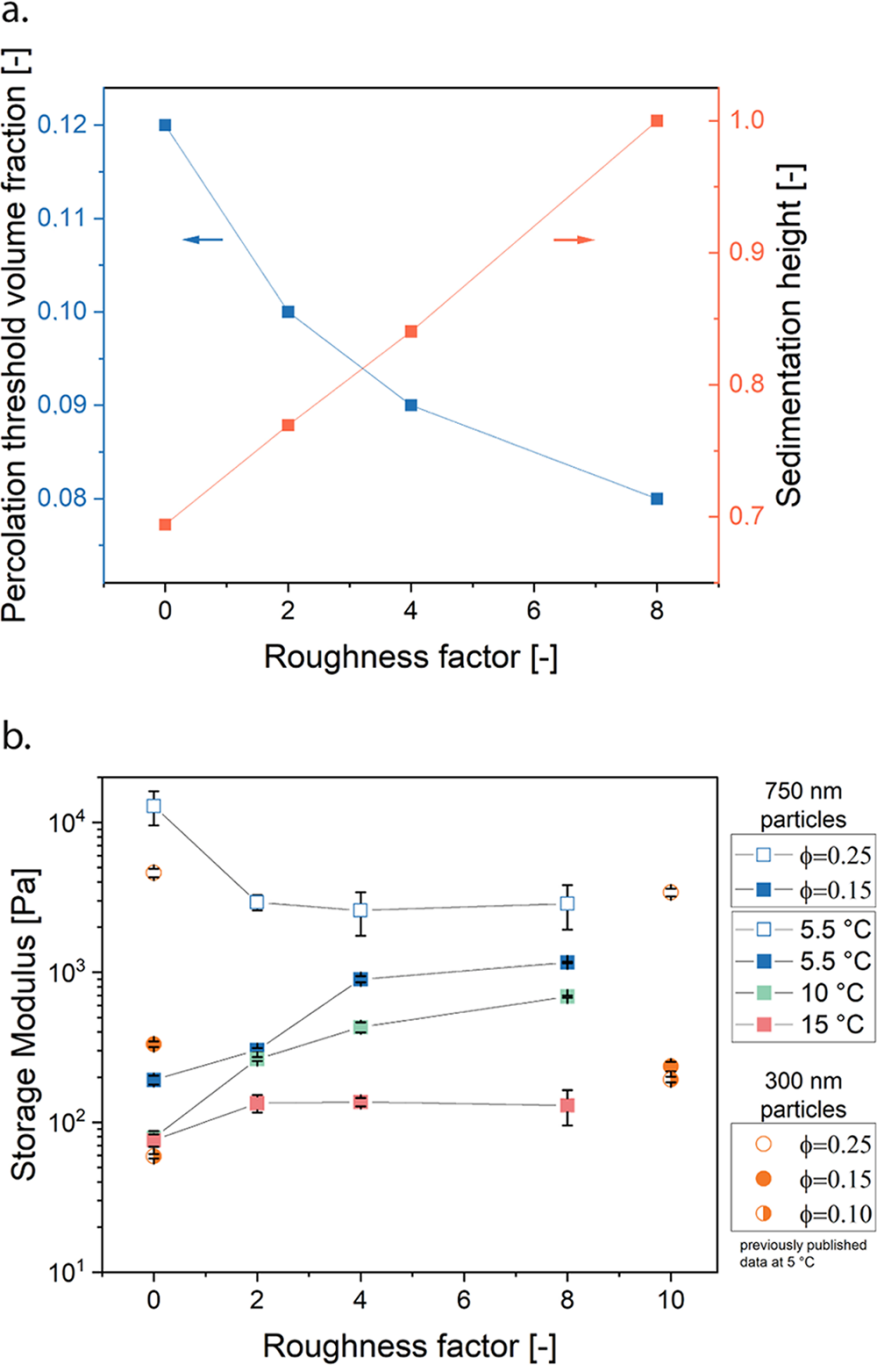
Quiescent bulk properties. (a) Percolation threshold and normalized sedimentation height (to *h*(*t* = 0)) for gels made out of particles of different roughness factors at a volume fraction ϕ = 0.15 and 5.5 °C, (b) Storage moduli of gels with varying particle roughness at volume fractions of ϕ = 0.15 (filled symbols) and ϕ = 0.25 (open symbols) across different temperatures. Squares represent measurements for particles with a core radius of 750 nm, while circles indicate previously published data for particles with a 300 nm radius, all measured at 5 °C.^28^ Error bars indicate the variability across three independent measurements.

In particular, the effects of roughness on adhesion and friction are reflected on the bulk properties of the suspensions and even more so in gels. As we have the unique capability to independently adjust both central and noncentral forces, this proves particularly insightful when constructing volume-spanning networks. Figure 3a reveals that, for gels formed by our particles at low temperature (high adhesion),^28,34^ percolation volume fraction at which samples showed a linear viscoelastic region where *G*′ > *G* “ over more than half a decade of strain amplitude. We conducted our experiments over more than half a decade of strain amplitudes, systematically increasing the volume fractions with each incremental step, using strain amplitude sweeps (see Materials and Methods). Percolation can be achieved with a lower volume fraction as the particle surfaces become rougher, showing that the percolation threshold in the gels is significantly reduced by making the particles rough. The fact that rough particles can interlock gives rise to rigidity in the colloidal network nodes, which hinders densification induced by gravitational flow, in turn reducing the needed particle loading for percolation. Gelation is a complex process, influenced by various factors. Recent research, such as the work conducted by Fenton et al.,^46^ suggests that gelation encompasses elements of percolation, phase separation, and glassy arrest. However, in the context of core–shell particle systems and at volume fractions like ours, percolation seems to be the dominant driving force for gelation, as demonstrated by Eberle and colleagues in their studies.^47,48^ Poon et al. emphasized the dependence of the percolation threshold on factors such as Brownian fluctuations (typically on the order of *k*_B_*T*), fractal dimension (*D*_f_), and, for density mismatched particles, the sedimentation kinetics^49^
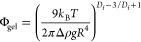
2were *k*_B_ is the Boltzmann constant, *T* is the temperature, Δ*ρ* is the difference in density between the particle and the suspending media, *g* is the gravitational constant and *R* is the particle radius. As we lower the temperature in relatively dilute systems, clusters are expected to begin to grow, and their fractal dimension, is determined by the speed of aggregation.^50^ Subsequently, these clusters start to aggregate to form a percolating network. However, it is important to note that clusters are also subjected to hydrodynamic forces, which may arise from thermal fluctuations or from settling effects, especially for a high density mismatch. When noncentral forces are absent, due to the presence of a rotational component (vorticity) in a simple shear flow, aggregates tend to undergo shear induced densification (increased *D*_f_), as it has been well documented in shear flows (see e.g., refs (51,52) and references therein) and in gravitational settling flows. This trend has also been observed in the percolation study conducted by Tsurusawa et al.,^53^ where clusters of smooth particles were found to compact due to the disturbance flow caused by gravitational settling before evolving into a volume-spanning network. Sedimentation kinetics, as illustrated in Figure 3a, hence plays a significant role in influencing the percolation threshold. Notably, the smooth particle gel at ϕ = 0.15 sedimented much faster (see Figure S10) than its rougher counterparts suggesting that the aggregates are more compact. Remarkably, the RF = 8 sample did not sediment at all over a period of 2 days. These observations align with the findings of Hsu et al., who established a direct correlation between the sliding friction coefficient of individual particles and the way these particle pack under gravity.^10^

Rheological characterization within the linear viscoelastic region for gels close to the percolation threshold, at ϕ = 0.15, is illustrated in Figure 3b (data refer to measurement points in the LVE region at steady temperature (15 min of equilibration) with complete strain amplitude sweeps available in Figure S6). Notably, as we increase the central forces (i.e., lower the temperature) the storage modulus exhibits a clear increasing trend, as previously reported.^34,50,54^ In our previous work, we have also reported that rough and smooth particle gels (particle core diameter = 300 nm) showed comparable storage moduli in the linear elastic regime throughout volume fractions of ϕ = 0.10 to ϕ = 0.25 (however, it is worth noting that at ϕ = 0.05 only a rough particle gel could be formed, as smooth particles did not percolate at ϕ = 0.05, but sedimented).^28^ It is important to consider that a volume fraction of ϕ = 0.15 is in close proximity to the percolation threshold, which inevitably impacts the overall network structure. Additionally, the larger size of the particles used in this study also increases gravitational effects. To investigate the extent to which surface roughness affects the quiescent gel properties, we conducted a comparison between gels at ϕ = 0.15 and ϕ = 0.25 (full and empty symbols in Figure 3b, respectively.). At ϕ = 0.25, for samples with RF = 2, RF = 4, and RF = 8, the storage modulus exhibited comparable values. However, the RF = 0 sample, characterized by smoother surfaces, displayed a higher storage modulus. For smaller particle gels, the role of surface roughness also appeared to influence the elastic response of the gel, however to a much lesser extent than for larger particle gels (see Figure 3b, yellow symbols). Notably, there is a scaling effect related to absolute particle size that influences the adhesion strength of rough particles across various sizes. The distance between the centers of mass of two interacting particles is proportional to the particle diameter, augmenting the particle size and adding asperities will increase the center-to-center distance even more, in turn decreasing central forces. Therefore, at higher particle loading (ϕ = 0.25, see Figure 3b empty symbols), the difference in elastic modulus between rough and smooth particle gels is greater for larger particles than it is for smaller ones. This difference can be furthermore attributed to the cumulative contact area between particles, which tends to be larger for smoother surfaces and consequently leads to a higher storage modulus. This is also reflected in the single-particle AFM measurements for adhesion in Figure 2c. This shows that for low volume fractions close to the percolation threshold, noncentral forces between the particles increase the elasticity of the gel. Meanwhile, for a more crowded system, the modulus is dominated by the total interacting surface area of the particles.

**Figure 4:**
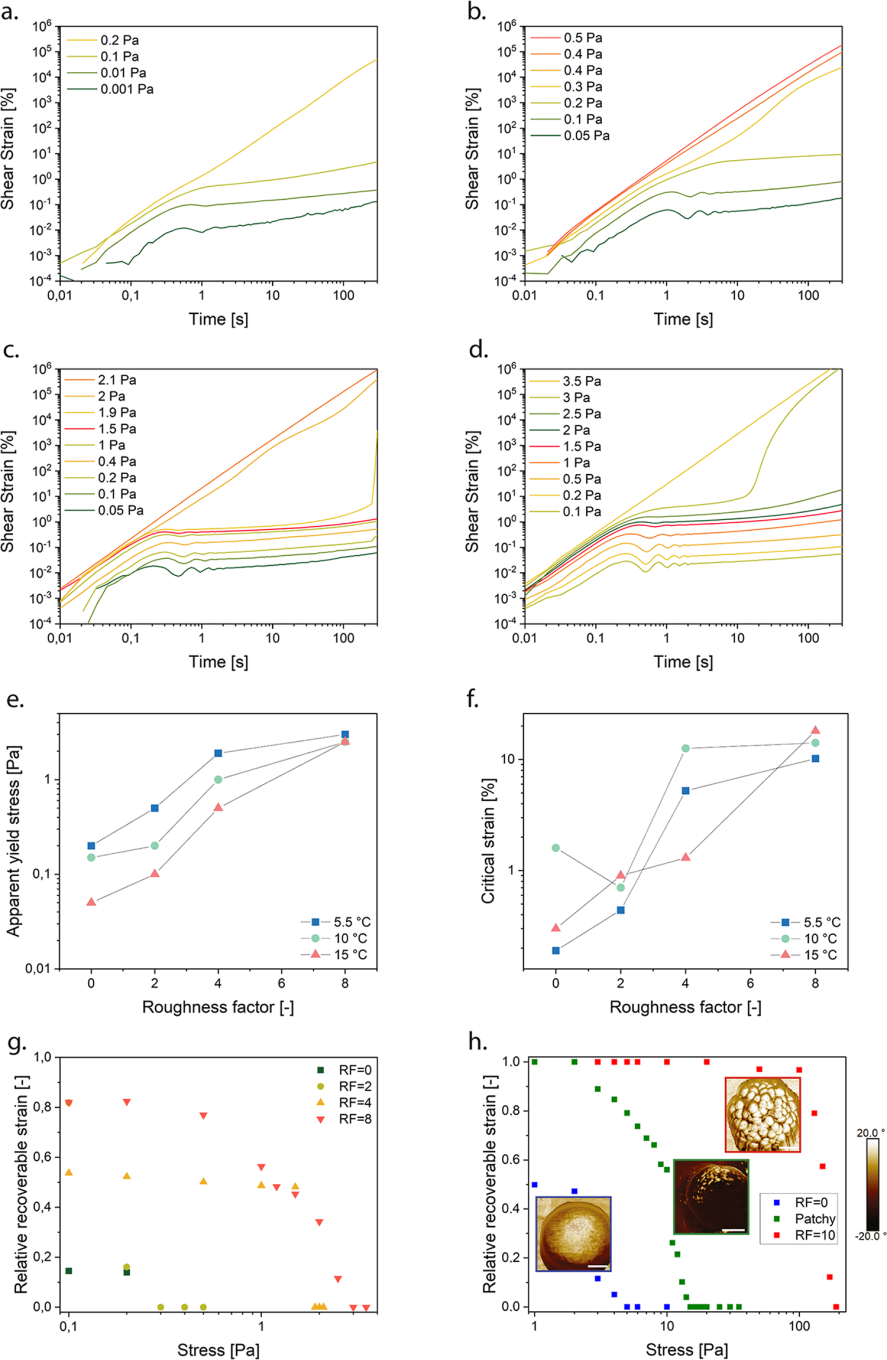
Nonlinear rheological properties. (a–d) Creep measurements of ϕ = 0.15 gels 5.5 °C at for roughness factors. (a) RF = 0, (b) RF = 2, (c) RF = 4, (d) RF = 8. (e) Apparent yield stress for gels with varying roughness and temperatures at volume fraction ϕ = 0.15. (f) Critical strain for gels with varying roughness and temperatures at volume fraction ϕ = 0.15. (g) Recoil measurements after the creep experiments for the different roughness at 5.5 °C.(h) Recoil measurements after creep for samples of a hydrodynamic radius of 300 nm for rough (RF = 10) and smooth (RF=0) particles^28^ as well as particles with a patchy distribution of octadecyl at 5.5 °C, where the insets show AFM phase images (positive phase shift indicates a softer surface - octadecyl - and a negative phase shift a harder surface - silica), showing a homogeneous octadecyl distribution on the smooth and rough particles and an inhomogeneous octadecyl coverage on the patchy particles (scale bars = 100 nm).

When subjecting the gelled samples to deformations beyond the linear elastic response regime, the samples yield and rearrangements are expected to occur, leading to a plastic flow or fracture. This behavior is observed in the creep experiments at maximal adhesion (*T* = 5.5 °C), as depicted in Figures 4a–d, for RF = 0 (a), RF = 2 (b), RF = 4 (c), and RF = 8 (d). In the creep experiment for smooth particles (Figure 3a), a gradual transition from a linear elastic region to fluidification begins to occur at a stress of 0.1 Pa. For RF = 2 (Figure 4b), the elastic region extends to higher values of the stress, showing elastic creep ringing until 0.1 Pa, transitioning to fluid-like behavior at 0.3 Pa. Figure 4c,d show the creep measurements for RF = 4 and RF = 8 gel samples, respectively, where the elastic region becomes even more prominent, and fluidification occurs abruptly, similar to fracture, at 1.9 Pa for RF = 4 and 3 Pa for RF = 8. (Creep experiments for varying roughness and temperatures are provided in Figure S7)

To gain a comprehensive view of the combined influence of central and noncentral forces, we summarized the apparent yield stress for all roughness factors (representing noncentral frictional forces) and temperatures (representing central adhesive forces) in Figure 4e, and the associated recoveries in Figure 4g. We mainly focused on the apparent yield stress and recovered strain, which increase with increasing surface roughness. However, a noteworthy trend emerges: the impact of central forces diminishes with higher roughness, suggesting that central forces become less influential on sufficiently rough surfaces. This is shown by the magnitude of the apparent yield stress in Figure 4e converging to the same temperature-independent value for RF = 8. Supporting this, the critical strain of the gels (Figure 4f, which was defined as the strain in the creep experiment where the slope first increases from the slope in the linear regime) correlates with roughness but not with temperature, indicating that noncentral forces dominate in determining deformation behavior as roughness increases.

Subsequently, the recovery following a 300 s long creep was also measured, reflecting the elasticity stored in the particle network. For smooth particle networks, elastic recoil decreases abruptly with applied stress, as plastic events and rearrangements occur readily. With increased roughness, the recoverable strain increases, indicating enhanced elastic energy can be stored more efficiently in the network and fewer plastic events occur (Figure 4g, with the full recoil measurements shown in Figure S8, and the relative recoverable strain for all RFs and temperatures in Figure S9). At equal concentration, and essentially unaltered adhesive strength, this points to a role of static friction in rigidity, as the networks become more resilient. Static friction constrains particle motion, leading to network structures constrained in nodes, akin to rigidity in bar frameworks, where a frictional node component increases network rigidity.^55,56^

To demonstrate this as a general concept, we designed geometrically smooth but chemically patchy particles, characterized the yielding behavior and recovery of gels made with these particles and compared them to smooth and maximally rough particle gels (Figure 4h, these particles all have a hydrodynamic radius of 300 nm, creep curves for patchy particles are shown in Figure S13). The evolution of the recovered strain of the patchy particles resembles the rough particle gels for low stresses, because the noncentral forces are still high enough to counteract the macroscopic shear. As the stresses increase, patchy particles will undergo local plastic events (similar to smooth particles) which will gradually decrease their ability to recover elastically. For rough particle gels, it is important to note that the elastic recovery is absolute, almost up until the application of the yield stress, upon which there is no more elastic recovery. Therefore, for the rough particle gels, there is a very abrupt elastic to plastic transition, which macroscopically shows static friction between particles and a homogeneous and abrupt fluidification upon the application of the yield stress. Understanding the occurrence of this instability depending on the nature of the interactions, and how stresses are distributed in the network will be the focus of further work.

## Conclusions

This study investigated the interplay of central and noncentral interparticle forces within colloidal particle gels, both at rest and during deformation. Using single-particle CP-AFM measurements, we quantified central (adhesive) and noncentral forces (friction) while independently controlling particle properties through adhesion force and surface roughness.

Our findings highlight the significant impact of surface roughness in reducing the percolation threshold of particle gels and influencing properties just above this threshold. The presence of static friction, generated by adhesion and resulting in noncentral forces, leads to network structures that are more easily constrained in nodes and can store elastic energy more resiliently. Building on prior research,^28^ our findings demonstrate that gels formed with rough particles exhibit a more uniform microstructure, leading to a more evenly distributed stress within the network. This structural homogeneity enhances gel toughness but also results in a more abrupt (brittle) yielding. The observed decoupling between toughness and ductility is a novel aspect that has not yet been extensively investigated. Traditionally, the transition from ductile to brittle behavior is primarily attributed to factors such as coordination number and local isostatic conditions.^31,32,57,58^ While these remain foundational concepts, they largely overlook the role of noncentral interactions and friction in the network nodes, which we suggest could be crucial in understanding the mechanical response of particle gels.

To further explore the concept of noncentral forces in particle gels, we synthesized geometrically smooth but chemically patchy particles and characterized the yielding behavior and recovery of gels made with these particles. The recoil experiments show, that a “chemical surface roughness” also enhances the elastic energy which can be stored in the particle network. Patchiness also induces noncentral interactions for low shear stresses, resulting in an enhanced elastic recovery as the nodes of the network become more resilient to deformation. If the shear stresses are increased, noncentral interparticle forces are overcome and plastic deformation occurs.

The results contribute to a deeper understanding of how tailored interparticle forces shape the behavior of complex colloidal systems. They underscore the potential for enhancing material efficiency in designing soft solids and colloidal gels by leveraging particle characteristics that hinder rotation, such as roughness and friction. This exploration is relevant for understanding industrial suspensions subjected to both gravitational and shear forces and developing new ways to control their rheology.

## Supporting Information Available

Additional experimental and characterization details including AFM calibration, CP-AFM force curves, friction measurements, rheological measurements, and patchy particle characterization (PDF)
