# Supplementary material for: Tuning Colloidal Gel Properties: The Influence of Central and Noncentral Forces
Source: Langmuir. 2025 Jan 28;41(5):3098–107. doi: 10.1021/acs.langmuir.4c03602 (PMC11823635; doi:10.1021/acs.langmuir.4c03602)
Supplement: Supplementary file 1 — la4c03602_si_001.pdf [file la4c03602_si_001.pdf]

Supporting Information: Tuning Colloidal Gel Properties: The Influence of Central and Non-Central Forces

Florence J. Müller[1], Shivaprakash N. Ramakrishna[1], Lucio Isa[1], and Jan Vermant[1]

[1]Department of Materials, ETH Zurich, 8093 Zurich, Switzerland,
jan.vermant@mat.ethz.ch

Number of pages: 15
Number of figures: 13
Number of tables: 1

Contents

1 Particle synthesis **S - 2**

2 Tribological characterization **S - 2**

3 Rheological characterization **S - 8**

4 Sedimentation characterization **S - 12**

1 Particle synthesis

Roughness factor	Mass of berry particles [g]	Volume of MAPTMS stock solution (5v%) [ml]	Volume of Octadecane-alkynoate stock solution (5w/v%) [ml]
RF=0	0	0.5	0.5
RF=2	0.02	0.51	0.53
RF=4	0.11	0.8	0.6
RF=8	0.18	0.85	0.7

Table S1: Proportions of the synthesis of the samples with different roughness factors.

2 Tribological characterization

AFM calibration

Normal and torsional spring constants were obtained from Sader's online calibration platform (https://ampc.ms.unimelb.edu.au/afm/calibration.html). The normal spring constant of all the cantilevers used are found to be 0.1 N/m and the torsional spring constants were found to be 5×10^{-9} Nm. The dimension of the cantilevers (length: 316 µm , width: 37 µm and thickness: 1.7 µm) were obtained from the scanning electron microscopy (SEM) imaging. To assess lateral thermal frequency and quality factors, we performed high-speed data capture at 6.25MHz for 500ms, using the lateral deflection channel as the output. The obtained deflection as a function of time was then converted into power spectra followed by fitting them to simple hormonic oscillator using a python script. The torsional sensitivity and the effective lateral spring constant values were obtained from equation 9 and 12 of Mullin et al. [4] respectively.

Johnson-Kendall-Roberts (JKR) Contact Model

The radius of the contact area a_c was calculated using JKR theory [1] chapter 17.

$$F_c = -3\gamma_{sv}\pi R \tag{S1}$$

with surface energy γ_{sv} and radius R. The contact area radius can then be calculated:

$$a_0 = (\frac{12\pi R^2\gamma_{sv}}{K})^{1/3}, \tag{S2}$$

where $K = 4/3E^*$ and E is defined as $1/E^* = 2\frac{1-\nu^2}{E}$ for two surfaces in contact of the same material [2]. In the calculations, the pull-off force F_c was taken from the adhesion measurements. Material properties for silicon-dioxide (silica) were used to calculate K ($\nu =$ 0.15, and $E = 66$ GPa, https://www.azom.com).

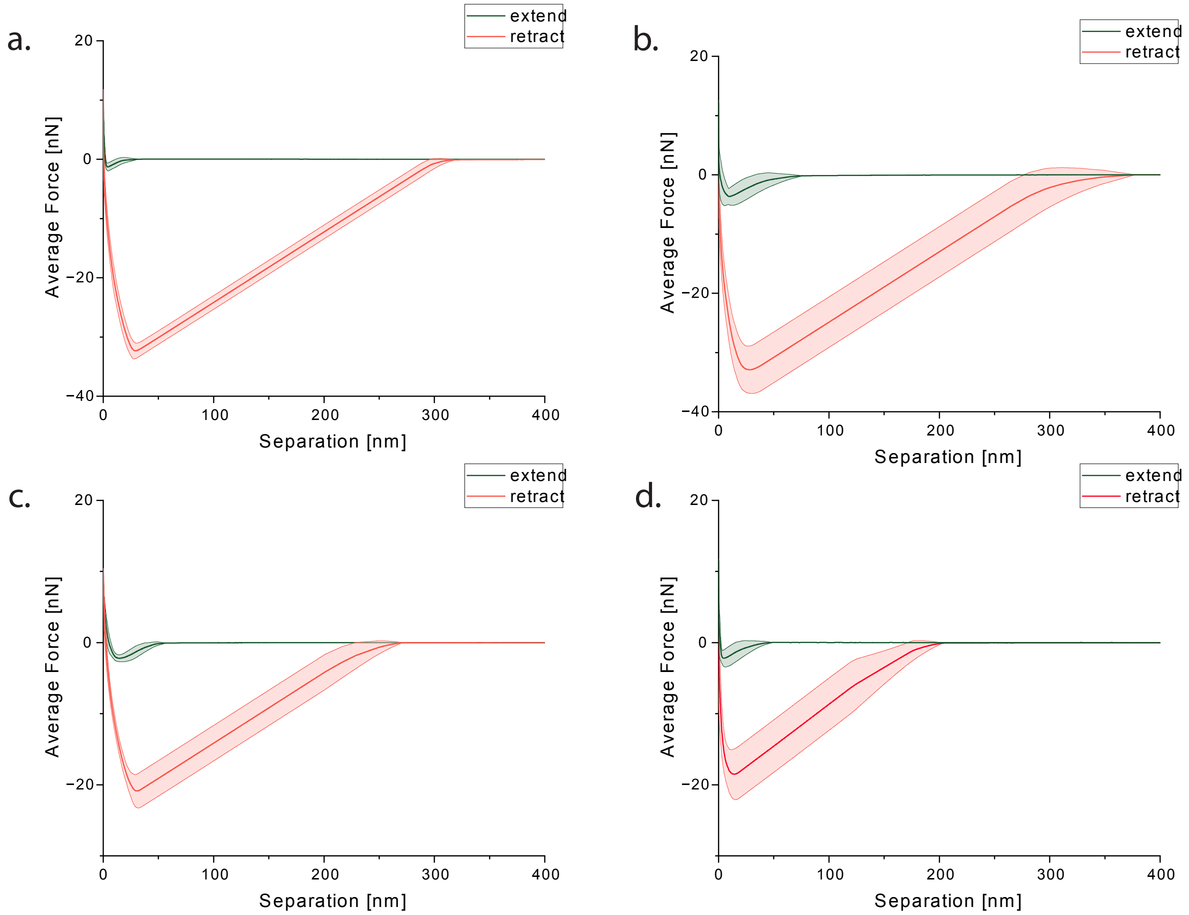

Figure S1: **Extend and retract adhesion force AFM colloidal probe experiments for** a. Smooth, b. RF=2, c. RF=4 and d. RF=8 particles at 5.5 °C

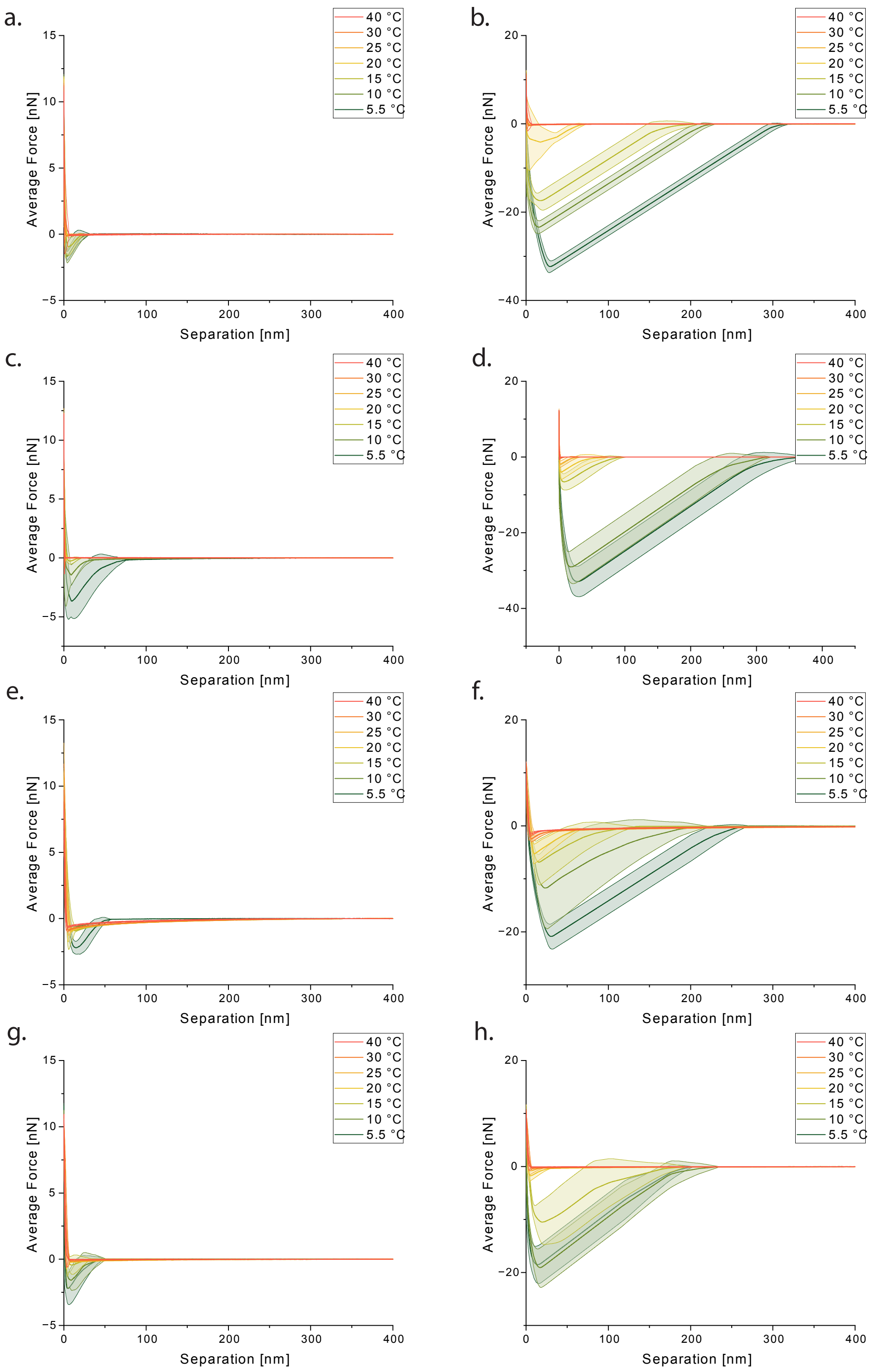

Figure S2: **Adhesion force AFM colloidal probe experiments** a. Smooth extend b. Smooth retract, c. RF=2 extend, d. RF=2 retract, e. RF=4 extend, f. RF=4 retract, g. RF=8 extend, h. RF=8 retract for different temperatures.

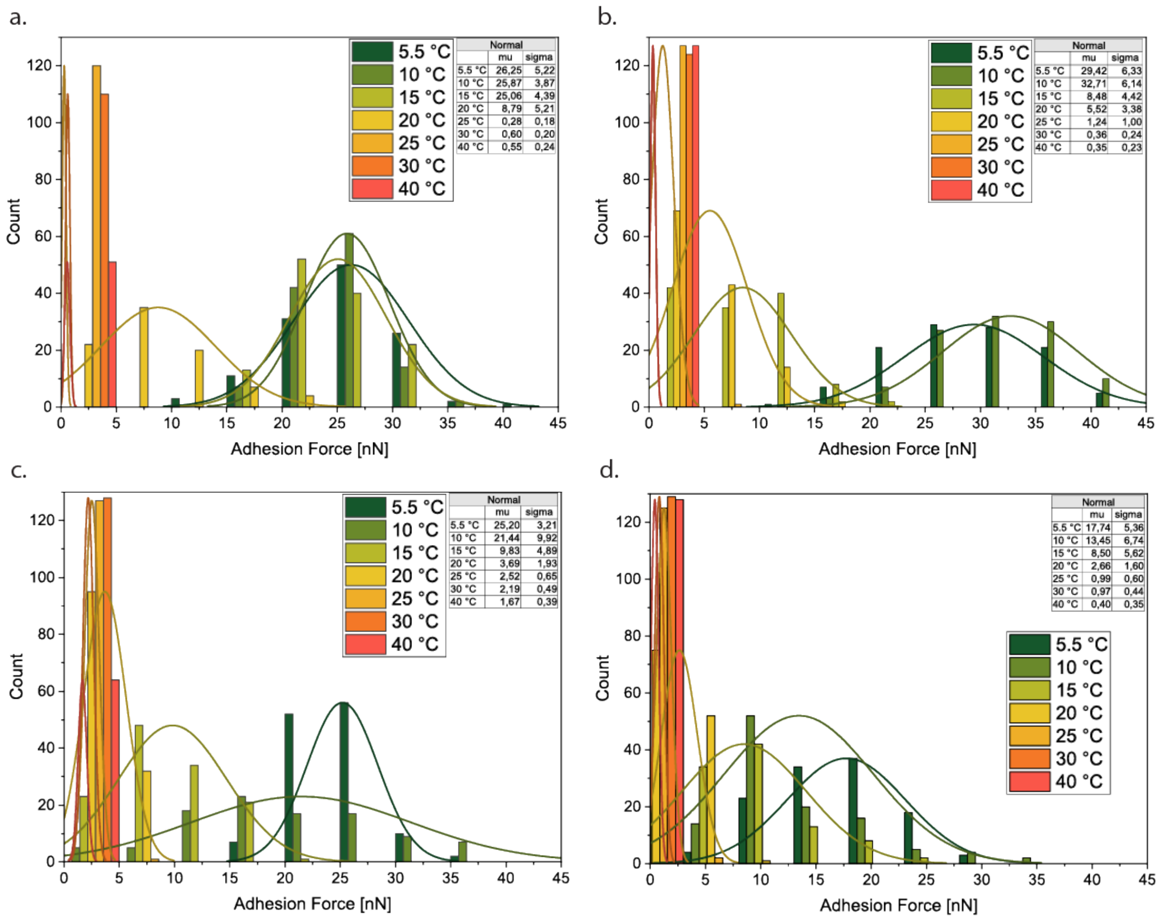

Figure S3: **Adhesion histograms for** a. Smooth, b. RF=2, c. RF=4 and d. RF=8 particles at different temperatures, fitted with a normal distribution.

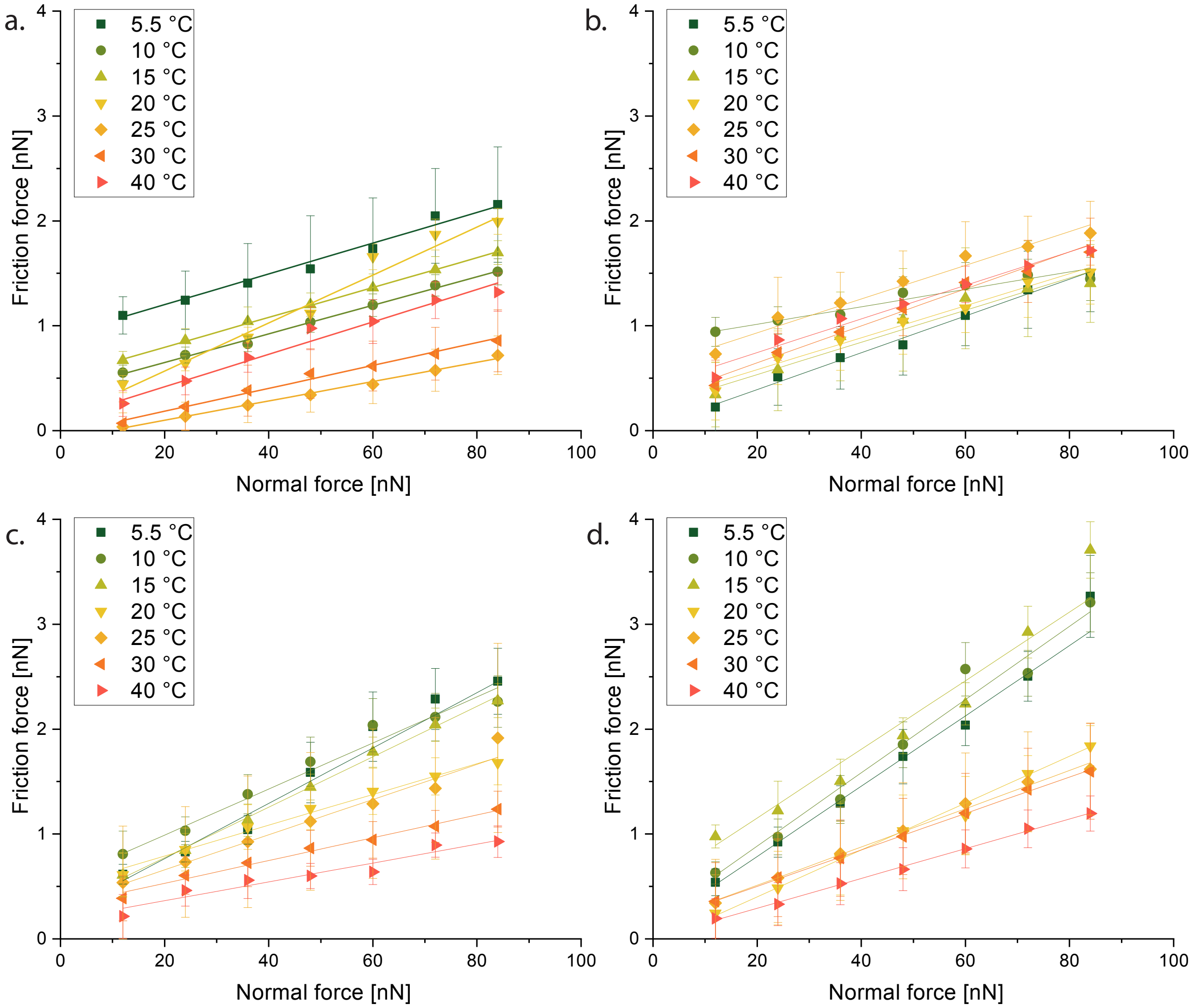

Figure S4: **Friction curves for** a. Smooth, b. RF=2, c. RF=4 and d. RF=8 particles at different temperatures.

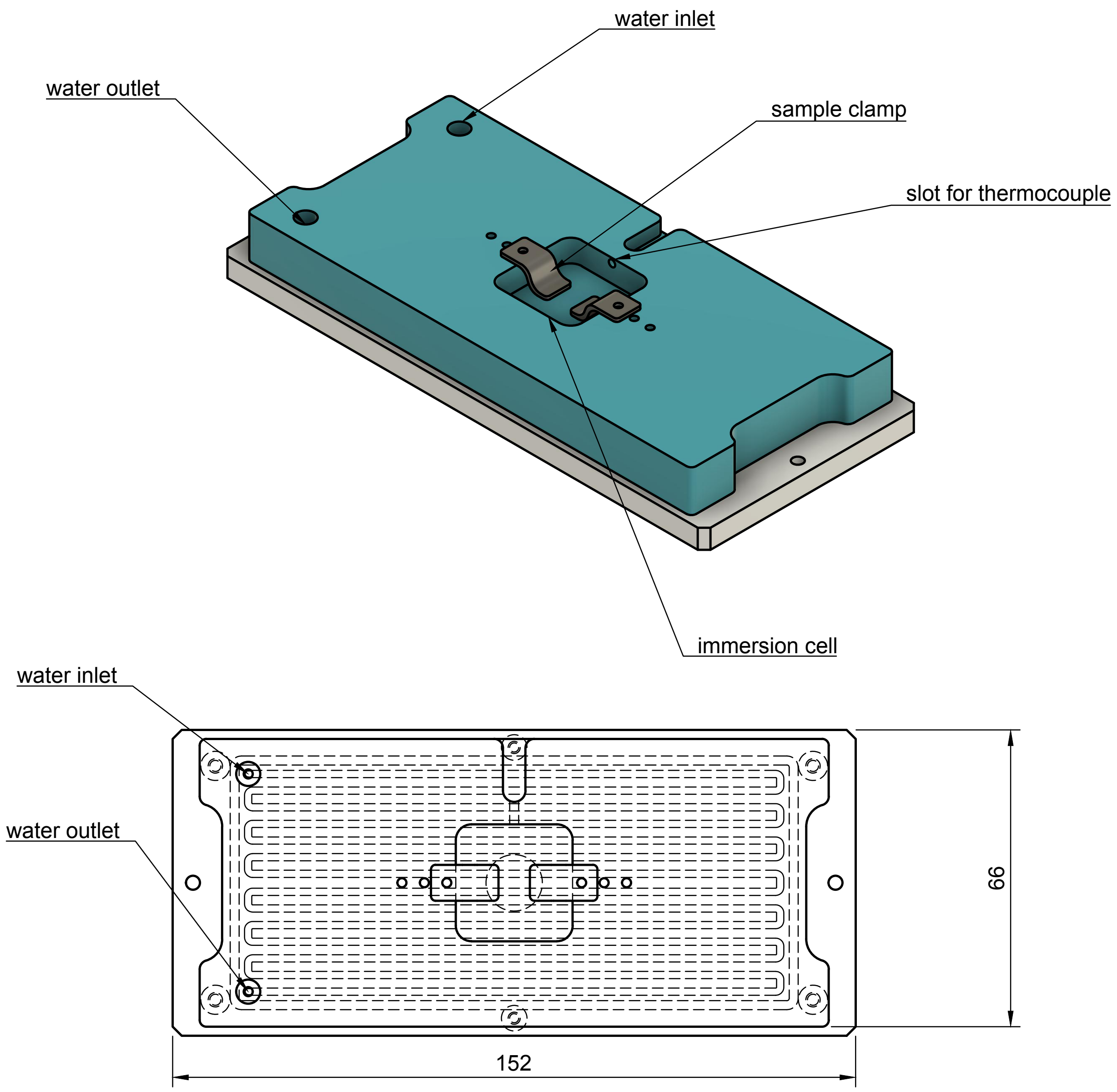

Figure S5: **Custom temperature cell (aluminium) with a secondary water cycle for temperature control, clamps to fix the substrate sample and an immersion cell for measurements in liquid.**

3 Rheological characterization

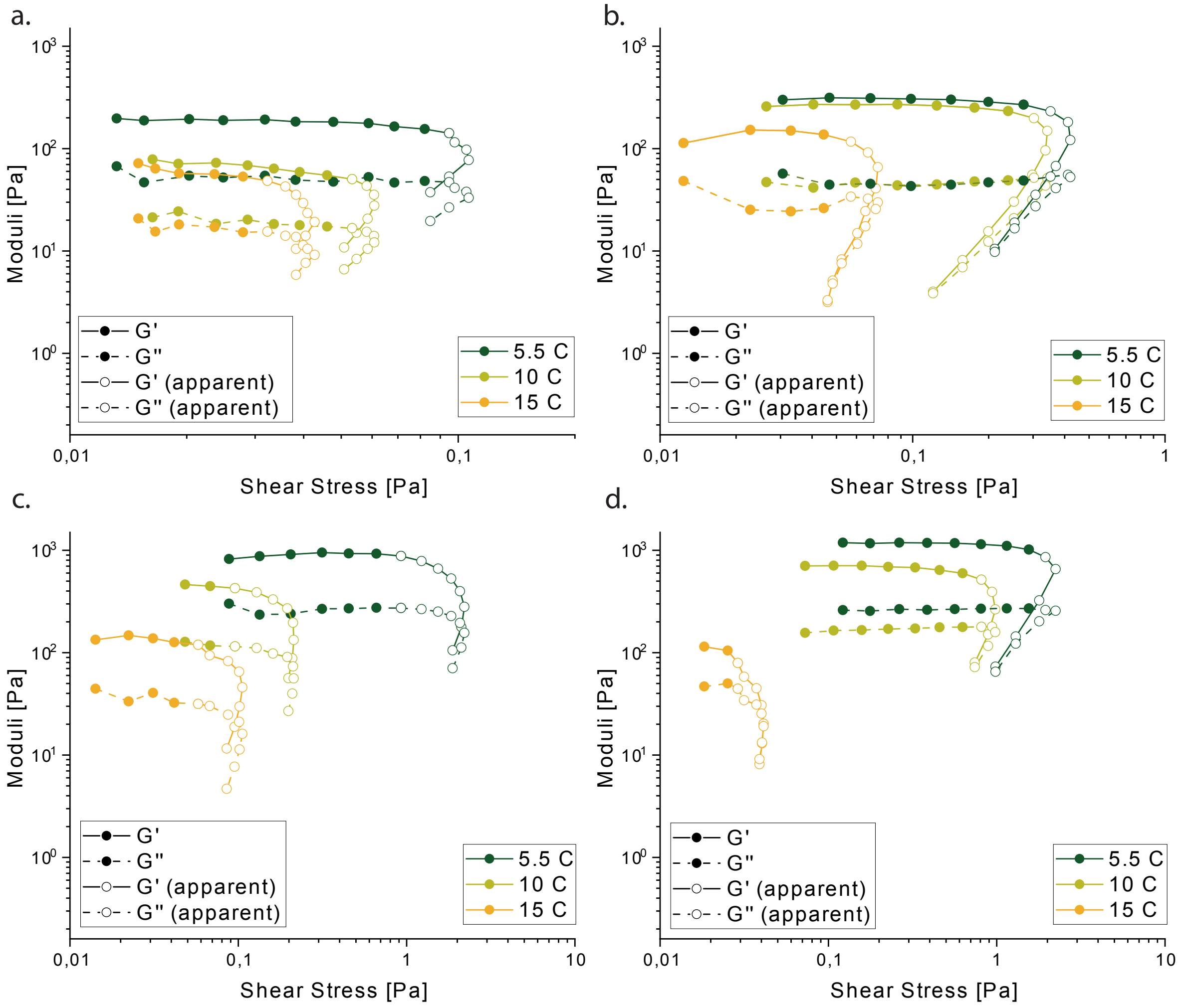

Figure S6: **Stress amplitude sweeps of ϕ=0.15 gels** a. Smooth, b. RF=2, c. RF=4 and d. RF=8 particles at 5.5 °C, 10 °C and 15 °C.

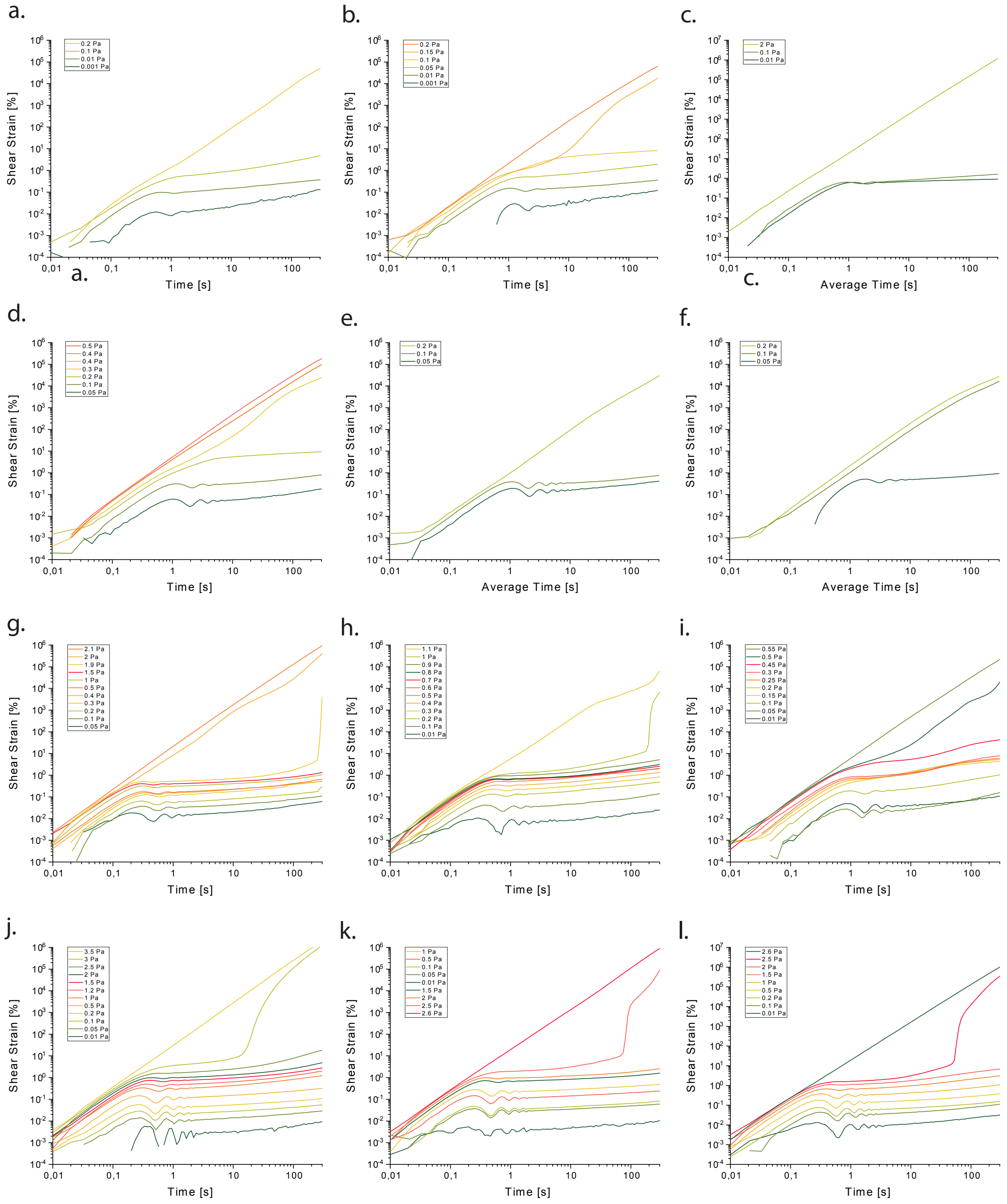

Figure S7: **Creep experiments for ϕ=0.15 gels** a. Smooth at 5.5 °C, b. Smooth at 10 °C, c. Smooth at 15 °C, d. RF=2 at 5.5 °C, e. RF=2 at 10 °C, f. RF=2 at 15 °C, g. RF=4 at 5.5 °C, h. RF=4 at 10 °C, i. RF=4 at 15 °C, j. RF=8 at 5.5 °C, k. RF=8 at 10 °C, l. RF=8 at 15 °C.

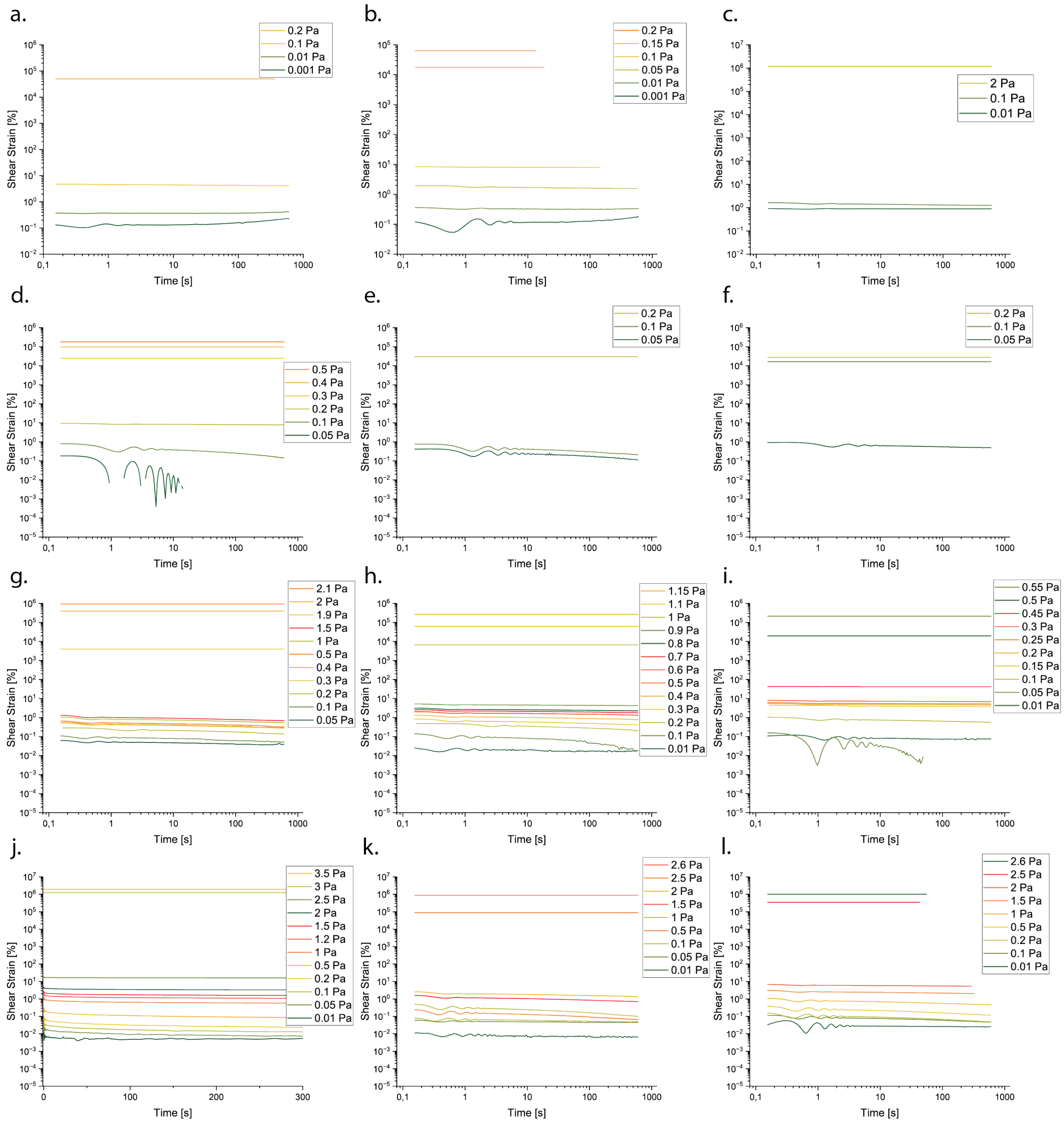

Figure S8: **Creep recovery experiments for ϕ=0.15 gels** a. Smooth at 5.5 °C, b. Smooth at 10 °C, c. Smooth at 15 °C, d. RF=2 at 5.5 °C, e. RF=2 at 10 °C, f. RF=2 at 15 °C, g. RF=4 at 5.5 °C, h. RF=4 at 10 °C, i. RF=4 at 15 °C, j. RF=8 at 5.5 °C, k. RF=8 at 10 °C, l. RF=8 at 15 °C.

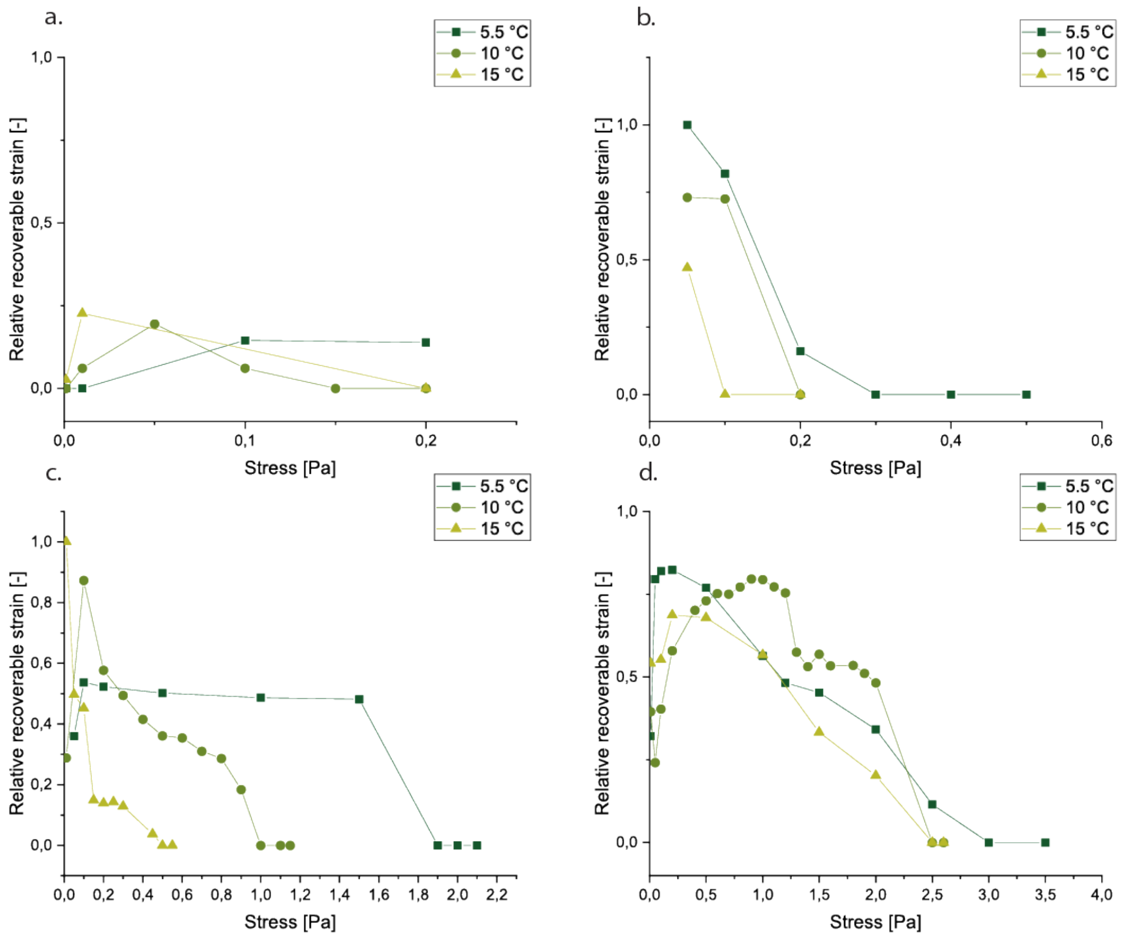

Figure S9: **Relative recoverable strain compared to total strain in the prior creep experiment of ϕ=0.15 gels** a. Smooth, b. RF=2, c. RF=4 and d. RF=8 particles at 5.5 °C, 10 °C and 15 °C.

4 Sedimentation characterization

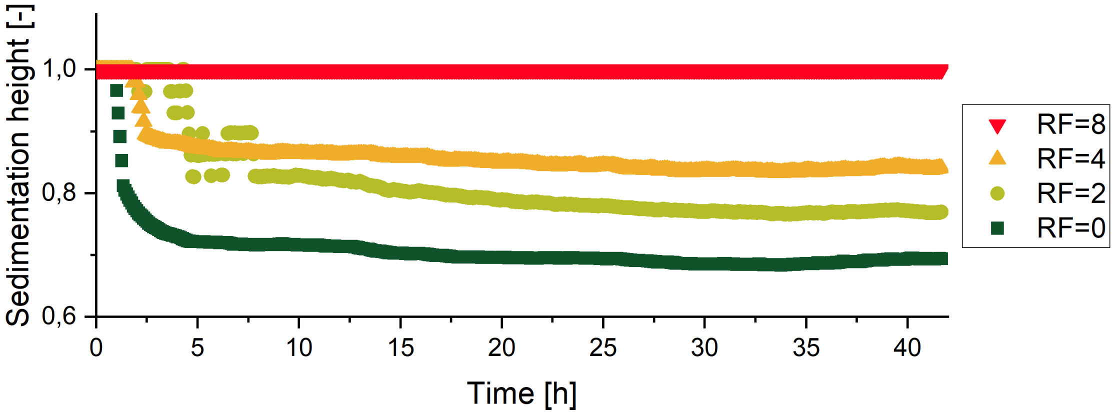

Figure S10: Normalized sedimentation height over time starting with ϕ=0.15 gels.

Patchy particle characterization

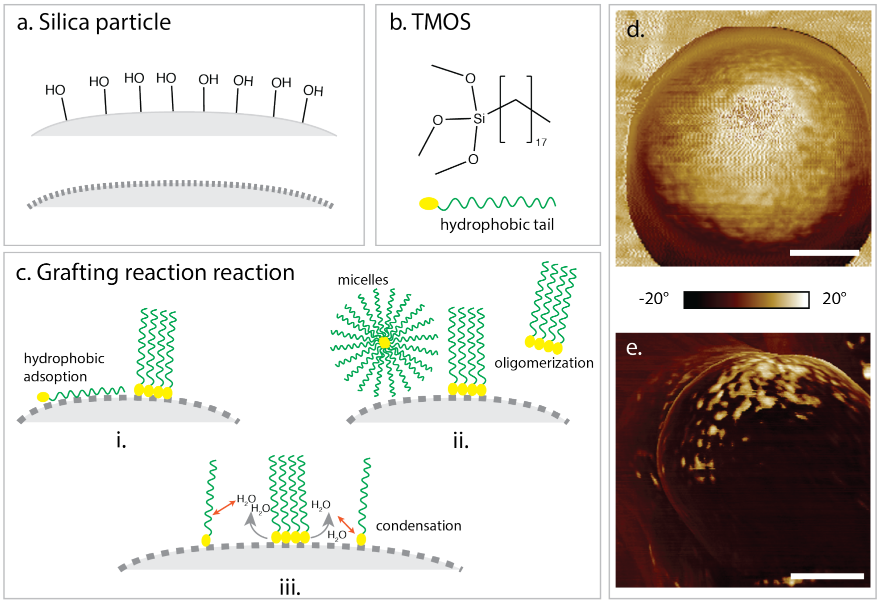

Figure S11: Synthesis and characterization of SiO_2-octadecyl patchy particles. a. Silica particle synthesized with the Stöber process showing hydroxyl surface groups, b. Amphiphilic triethyl(octadecyl)silane (TMOS) grafting agent, c. Condensation reaction of TMOS to the surface of the particle in a patchy surface coverage due to the hydrophopic tail, d. AFM phase imaging for the homogeneously grafted silica particles using click chemistry [3], e. AFM phase imaging for the patchy grafted particles where the bright spots indicate the TMOS and the darker spots indicate the harder silica. The scale bars correspond to 100 nm.

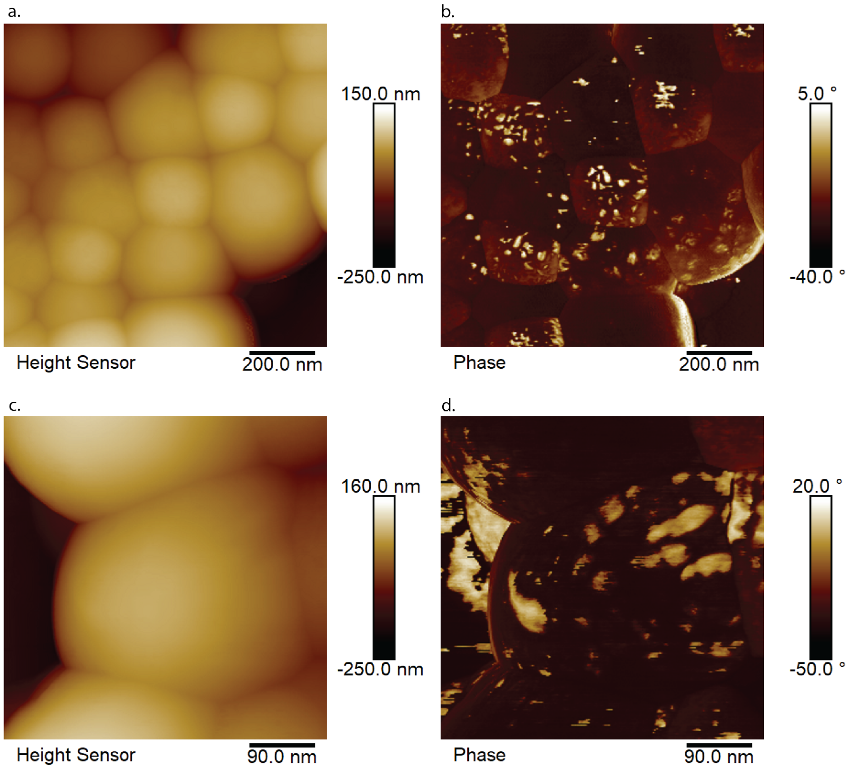

Figure S12: AFM measurement of patchy particles. a. Height sensor, b. Phase imaging, c. Height sensor, d. Phase imaging

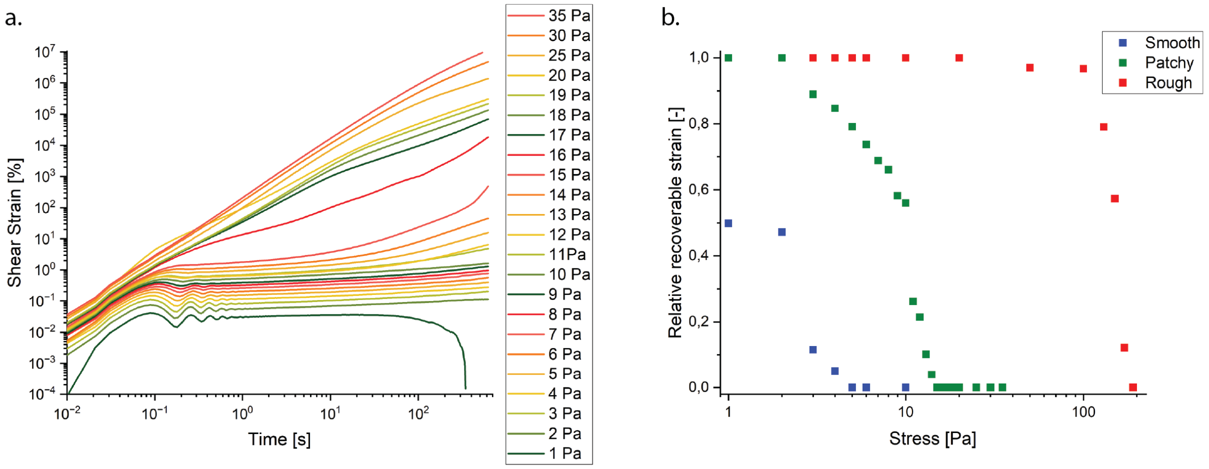

Figure S13: Creep and recovery experiments for the patchy particles at ϕ=0.25 and 5.5 °C. a. Creep experiment of the patchy particles, where the sample shows a rough-like behavior for low stresses and a smooth-like behavior for high stresses, b. Elastic recovery after the creep stresses for the smooth, rough [3] and patchy particles.

References

[1] Jacob B. Israelachvili. *Intermolecular and surface forces*. Elsevier, 2011.

[2] Ioan D. Marinescu et al. “4 - Contact Mechanics”. In: *Tribology of Abrasive Machining Processes*. Ed. by Ioan D. Marinescu et al. Norwich, NY: William Andrew Publishing, 2004, pp. 91–119. ISBN: 978-0-8155-1490-9. DOI: https://doi.org/10.1016/B978-081551490-9.50005-0. URL: https://www.sciencedirect.com/science/article/pii/B9780815514909500050.

[3] F. J. Müller, L. Isa, and J. Vermant. “Toughening colloidal gels using rough building blocks”. In: *Nat Commun* 14.1 (2023), p. 5309. ISSN: 2041-1723 (Electronic) 2041-1723 (Linking). DOI: 10.1038/s41467-023-41098-9. URL: https://www.ncbi.nlm.nih.gov/pubmed/37652918.

[4] Nic Mullin and Jamie K Hobbs. “A non-contact, thermal noise based method for the calibration of lateral deflection sensitivity in atomic force microscopy”. In: *Review of Scientific Instruments* 85.11 (2014).
